# Supplementary material for: Wheat Yellow Mosaic Virus NIb Interacting with Host Light Induced Protein (LIP) Facilitates Its Infection through Perturbing the Abscisic Acid Pathway in Wheat
Source: Biology (Basel). 2019 Oct 23;8(4):80. doi: 10.3390/biology8040080 (PMC6955802; doi:10.3390/biology8040080)
Supplement: Supplementary file 1 [file biology-08-00080-s001.zip › biology-599420-supplement Fig S1, S2,Tab S1.docx]

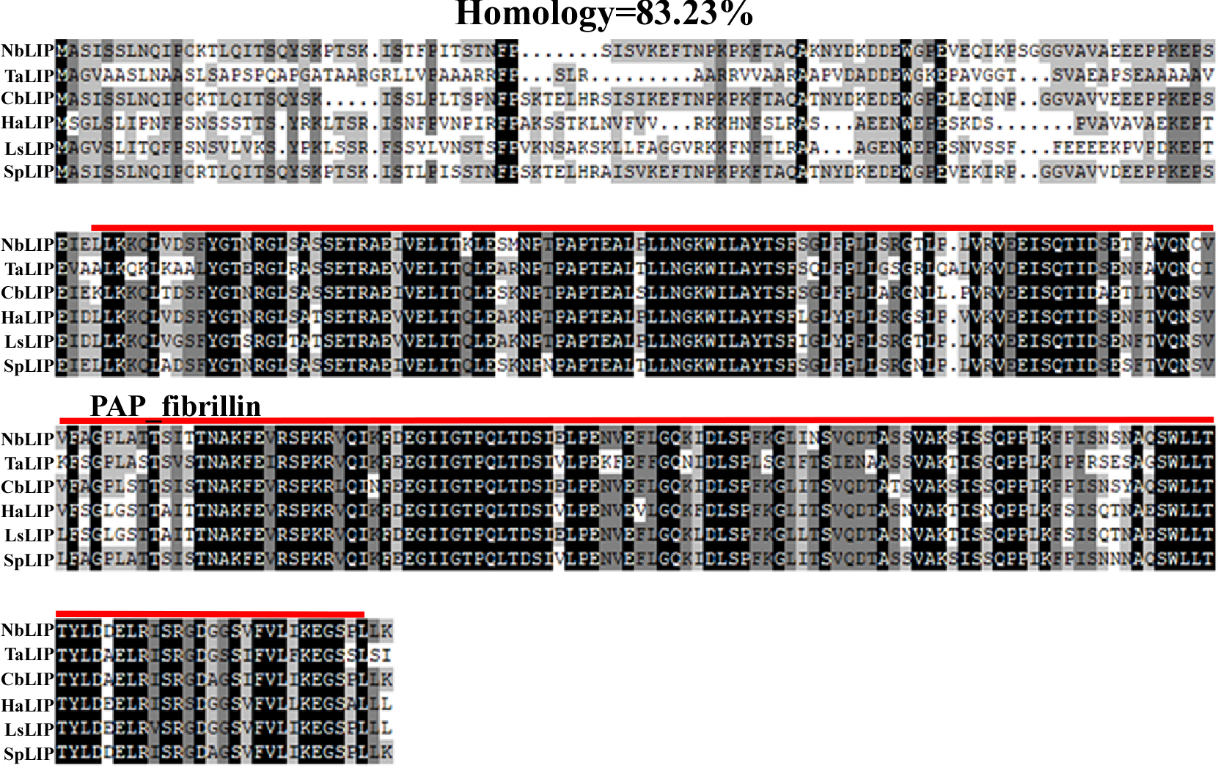


**Supplementary Figure S1.** Sequence homology alignment between the LIP homologous genes of six different species. *Nb* (*Nicotiana benthamiana*), *Ta* (*Triticum aestivum*), *Cb* (*Capsicum baccatum*), *Ha* (*Helianthus annuus*), *Ls* (*Lactuca sativa*), *Sp* (*Solanum pennellii*). The red line indicates the conserved domain of proteins belonging to the fibrillin family.


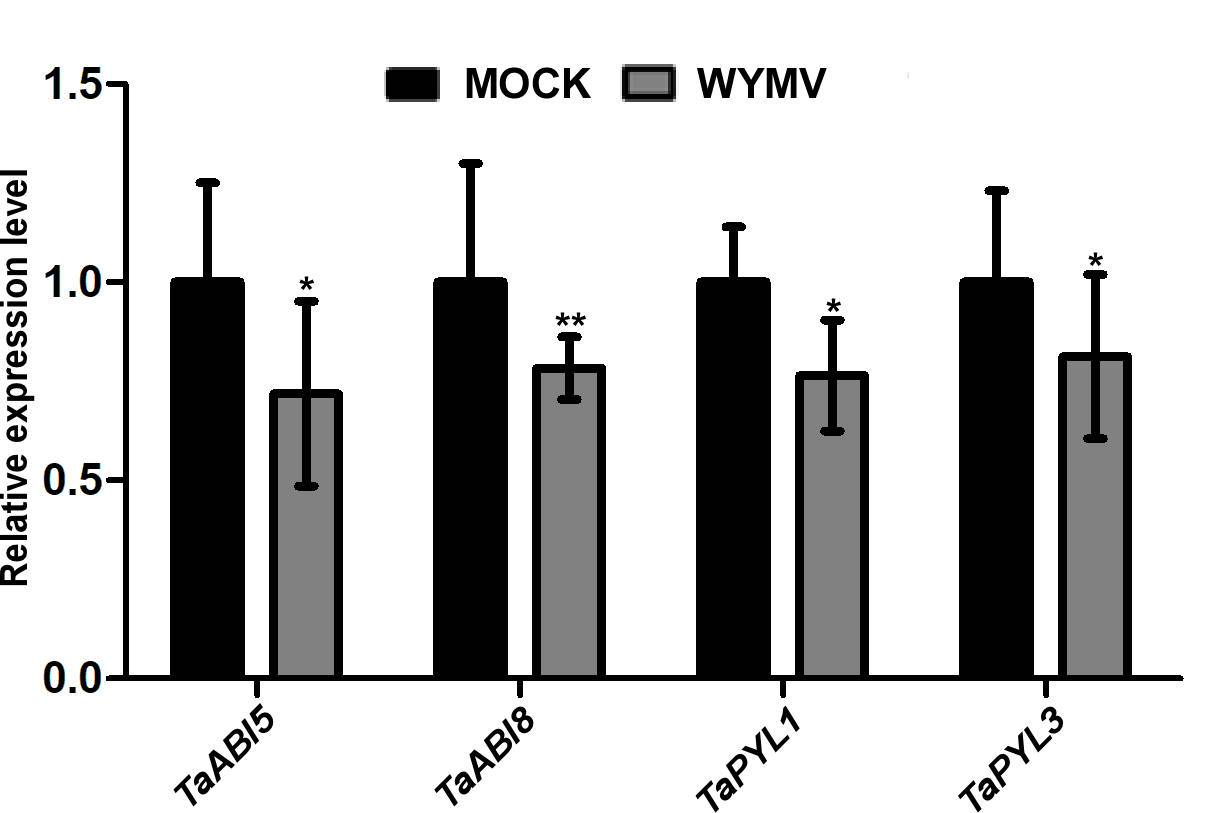


**Supplementary Figure S2.** Transcriptome analysis of WYMV-infected wheat. Healthy wheat was used as the negative control. Each relative expression level is presented as the mean ± SD from six biological samples and each biological sample had four technical replicates. Statistical analyses were done using the Student’s *t*-test. Asterisks indicate significant difference when compared to the control. * *p* < 0.05; ** *p* < 0.01.

**Supplementary Table S1.** Information of proteins list obtained from Yeast two-hybrid assay.

| **NAME** | **LOC** | **ORF** | **CLONE** |
| --- | --- | --- | --- |
| 1.chlorophyll a-b binding protein 50 | XM_016590181 | FULL | 2 |
| 2.Phosphomethylpyrimidine synthase | XP_009772632 | fragment | 2 |
| 3.light-induced protein | AK454210.1 | fragment | 8 |
| 4.GDP-L-galactose, phosphorylase | XM_016651960 | FULL | 5 |
| 5.Chlorophyll a-b binding protein 40 | XP_009795316 | FULL | 2 |
| 6.pupal cuticle protein | XP_009762243 | fragment | 1 |
